# Effiformer: a unified data-efficient vision transformer-CNN framework for interpretable epileptic seizure detection

**DOI:** 10.1186/s40708-026-00320-2

**Published:** 2026-07-21

**Authors:** Aaranay Aadi, Divyansh Sukhija, Rishabh Shetty, Praveen Shukla, Vijaypal Singh Dhaka

**Affiliations:** 1 School of Computer Science and Engineering, Faculty of Science, Technology and Architecure (FoSTA), Manipal University Jaipur, Jaipur, Rajasthan 303007 India; 2 Department of IoT and Intelligent Systems, School of Computer Science and Engineering, Faculty of Science, Technology and Architecure (FoSTA), Manipal University Jaipur, Jaipur, Rajasthan 303007 India; 3 Department of Computer and Communication Engineering, School of Computer Science and Engineering, Faculty of Science, Technology and Architecure (FoSTA), Manipal University Jaipur, Jaipur, Rajasthan 303007 India

**Keywords:** Deep learning, Electroencaphelogram (EEG), Epileptic seizure, Vision transformer (ViT), Rehabilitation, Health Disparity

## Abstract

Epileptic seizures are short episodes of abnormal electrical activity in the brain that can cause convulsions, loss of consciousness, and other similar symptoms. Despite therapy, around 30% of patients with epilepsy continue to have seizures, emphasizing the necessity for quick and effective detection measures. Accurate seizure detection allows for prompt intervention, which dramatically improves patient safety. This study has developed EffiFormer, a hybrid Vision Transformer-CNN model for reliable seizure detection using Electroencaphelogram (EEG) spectrograms. EffiFormer combines spatial feature extraction from EfficientNet with global attention mechanisms from the Data-Efficient Image Transformer, resulting in high accuracy even with limited training data. Our four-phase pipeline processes raw EEG signals through normalization, Short-Time Fourier Transform (STFT) to create spectrograms, synthetic seizure sample generation using SMOTE, and data augmentation. This study utilized a standard 60-20-20 training-validation-testing split for effectively training our proposed architecture and validating its accuracy. Further, this study tests the technique using the CHB-MIT dataset, which is a publicly available collection of long-term scalp EEG recordings from 22 pediatric patients at Children’s Hospital Boston and covers a wide spectrum of seizure and non-seizure episodes. With an average sensitivity of 99.8% and average accuracy of 99.3%, our approach offers accurate seizure detection in various scenarios. Furthermore, explainable AI (XAI) approaches emphasize EEG regions that influence the model’s results, making the model’s decision-making process more transparent and interpretable for potential clinical review.

## Introduction

### Problem overview

Epilepsy is a neurological disorder characterized by recurrent seizures from abnormal brain activity. Symptoms commonly include sudden, unpredictable convulsive movements, brief auras (such as sensory disturbances and tingling), loss of consciousness, autonomic changes, and cognitive or behavioral alterations [1]. Its clinical manifestations vary by type, including focal onset seizures (which begin in one area of the brain), generalized onset seizures (which affect both sides of the brain from the start, such as absence and tonic–clonic seizures), and unknown onset seizures (where the beginning of the seizure is not observed or unclear) [2]. The World Health Organization (WHO) estimates that 50 million individuals worldwide suffer with epilepsy, making it one of the most prevalent neurological conditions. Besides the physical symptoms, epilepsy also compromises mental health, quality of life, and can lead to severe complications or death [3, 4]. Although anti-epileptic drugs are the most common treatment, nearly 30% of patients remain drug-resistant [5], making the accurate detection of seizure an essential need.

Electroencaphelogram (EEG) signals remain one of the best tools for seizure detection. EEG recordings can be categorized into different phases: the preictal phase (the period before a seizure), the ictal phase (the actual seizure event), the postictal phase (the recovery period following a seizure), and the interictal phase (the time between seizures when the brain appears to function normally) [6–8]. These recordings have long been used to identify seizures since they reflect the firing of electrical impulses by a group of neurons. [9]. However, manual review of EEG data is inefficient, slow, and is based more on experience [10, 11]. That is why detection methods have emerged using techniques like the Short-Time Fourier Transform (STFT) [12], Wavelet Transform (WT) [13], Wigner-Ville Distribution (WVD) [14], and Welch’s method [15, 16]. The evolution of seizure detection has moved from feature engineering using approaches like Fourier analysis and empirical mode decomposition [17] to data-driven deep learning methods.

### Related works

Early machine learning architectures, such as support vector machines, decision trees, and random forests [18] depended heavily on select features but struggled with the EEG patterns which were non-linear and did not fit a typical pattern.

Deep learning architectures, such as CNNs, RNNs, and LSTMs can do feature extraction and directly process raw EEG data, which significantly improves accuracy and makes the pipeline simple [19–22]. Transformer-based models use self-attention to capture long-range dependencies and contextual relationships within EEG signals [23–25]. Unsupervised transformers work by solving the problem as an anomaly detection problem [26]. Despite these advances, some challenges remain. Deep learning models require large and well-annotated datasets across patients. In addition to that, transformer-based methods face issues like high computational complexity and are very black box in nature. The rapid evolution of methods starting from basic Feature Based Methods to Deep Learning Methods like Transformers represents the latent potential.

Transformer-based methods have improved EEG seizure diagnosis by capturing long-range temporal and spatial features. Zhu et al. [27] introduced transformer models for multi-channel EEG, using self-attention to highlight key signal patterns while addressing computational overhead and generalizability. Ke et al. [28] proposed convolutional transformer networks that combine convolutional spatial feature extraction with transformer-based temporal modeling, showing improved performance on standard datasets. Yuanliu et al. [29] showcased hybrid approaches with a deep C-LSTM network that combines sequential models with attention mechanisms. Vision transformer (ViT) architectures have also been explored; for instance, Yuan et al. [30] developed a DenseNet-ViT hybrid that effectively models spatial and temporal EEG dynamics, and Chen et al. [31] introduced a spiking convolutional transformer to improve temporal resolution with lower computational cost. Hybrid architectures that combine LSTMs and transformers are another focus. Xia et al. [32] used LSTMs for short-term dependencies along with transformers for long-term dependencies, while Chiranjeevi and Keerthana [33] and Wang et al. [34] highlighted real-time detection challenges and trade-offs in transformers. Other studies have broadened transformer applications in seizure detection. Hussein and Lee [35] and Zhang and Li [36] used vision transformers for multichannel and patient-specific predictions, despite issues with generalizability and data requirements. Yan et al. [37] and later Li [38] and Chen and Parhi [39] applied transformers and CNN-transformer hybrids directly to scalp EEG data, though interpretability remains challenging. Innovative extensions include Hireche and Sirpal’s [40] use of vision transformers for decoding seizure patterns, Shi and Li’s [41] B2-ViT Net with broad attention, and Parani et al.’s [42] integration of pretrained vision transformers with language models for transfer learning across domains.

Despite the substantial progress in EEG-based seizure detection using deep learning and transformer-based architectures, several limitations persist across existing approaches. First, while transformer models effectively capture long-range dependencies, their high computational complexity limits their applicability in real-time or resource-constrained clinical settings. Second, many models demonstrate strong performance on benchmark datasets but struggle to generalize across patients due to inter-subject variability in EEG signals. Third, although hybrid architectures attempt to balance spatial and temporal feature extraction, they often still rely heavily on large annotated datasets, which are scarce in medical domains. Finally, the interpretability of these models remains limited, as most approaches function as black-box systems, making it difficult for clinicians to trust and validate their predictions.

These limitations highlight a critical research gap: the need for a computationally efficient, generalizable, and interpretable framework that can operate effectively on limited labeled EEG data while still leveraging the strengths of transformer-based architectures.

### Contribution

The limitations identified in the literature, namely high computational complexity, poor cross-patient generalization, data dependency, and lack of interpretability, motivate the following key contributions of this work:

- *Hybrid CNN–Transformer Architecture for Efficiency and Representation Learning:* This study proposes a unified architecture that combines a convolutional neural network (EfficientNet) with a vision transformer (DeiT-III). The CNN captures localized time-frequency patterns, while the transformer models long-range dependencies, resulting in a computationally efficient yet expressive model.
- *Time-Frequency Reformulation of EEG Signals:* Raw EEG signals are transformed into spectrogram representations, enabling seizure detection to be framed as an image-based learning problem. This transformation is clinically motivated in a conceptual sense, as epileptic seizures often manifest as structured patterns in the time-frequency domain, including rhythmic discharges, frequency shifts, and transient energy bursts that may be difficult to capture in raw signal space.
- *Improved Generalization and Data Efficiency:* To address limited labeled data and inter-patient variability, the proposed framework integrates self-supervised learning, data augmentation, and *runtime* oversampling (as opposed to static dataset expansion). This improves feature learning from small samples while mitigating class imbalance and enhancing generalization across patients.
- *Lightweight Design for Scalable Deployment:* Unlike conventional deep learning approaches that require large-scale training data and high computational resources, the proposed hybrid model achieves strong performance with a relatively compact parameter space, making it more suitable for resource-constrained environments.
- *Interpretability via Time-Frequency Localization:* To address the black-box nature of deep models, a post-hoc interpretability framework is introduced that operates at the feature level in the time-frequency domain. Unlike standard attention-based explanations, which may not reliably reflect causal importance, the proposed method highlights clinically relevant regions associated with seizure activity. This enables neurologists to distinguish true ictal patterns from artifacts, supporting model transparency and potential clinical applications.

## Materials and methods

**Fig. 1:**
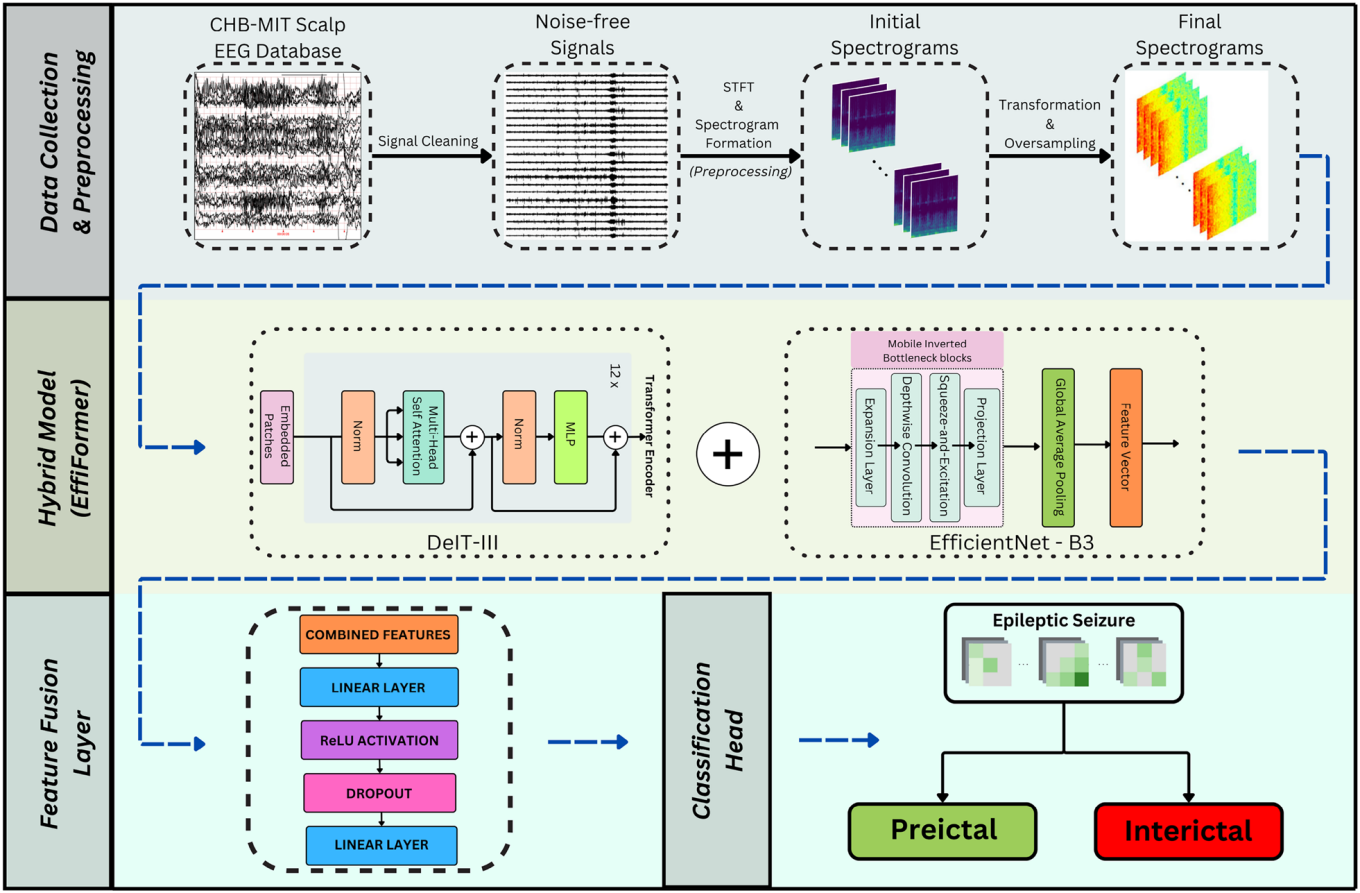
Block diagram of the proposed methodology with (1) representing data gathering and initial preprocessing, (2) visualizing the summarized architecture of the hybrid model, (3) showing the feature fusion process, and (4) representing the final classification output

The proposed methodology for seizure detection, encompassing data collection, preprocessing, hybrid model architecture, feature fusion, and final classification is illustrated in Fig. 1 and explained in the subsequent subsections.

### Dataset description

This study utilised the CHB-MIT Dataset [43–45] for EEG data. This dataset is publicly accessible and it was created by MIT and Boston Children’s Hospital. It provides high-resolution scalp EEG recordings from 23 children with epilepsy. As such, it is a popular tool for seizure detection research. In order to capture minute seizure activity, the dataset uses a 256 Hz sampling rate and adheres to stringent clinical procedures. EEG channels range from 9 to 23 per recording, with CZ-PZ, FZ-CZ, P8-O2, T8-P2, F8-FP2, P4-C4, and C3-F3 being some of the most common channels. About 972 h of EEG recordings format are included, with accurate seizure start and offset annotations provided by expert neurologists. There are 198 total seizure events in the entire dataset, across 24 patients. The EEG data for preictal conditions are obtained 30 min before the seizure. Between the start of the seizure and the preictal period, there is approximately one minute for intervention. The time frame is hanging before and after the beginning of the interictal seizure.

In order to mitigate overfitting, the dataset includes individuals who experienced at least three consecutive seizures within a three-hour period. This guarantees relevance in therapy by providing real-world epilepsy diagnostic and treatment issues. Within the dataset, 85 seizure occurrences are recorded in total. Because of its extensive annotations, ethical considerations, and public accessibility, CHB-MIT dataset is a vital tool for creating reliable seizure detection and prediction models.

### Pre-processing

Preprocessing is a necessary step in the pipeline. To improve the process a three stage pipeline is undertaken to ensure best possible spectrograms are created.

#### Signal cleaning

EEG signals have to go through the necessary filtering and augmentation procedures during preprocessing. To eliminate electrical source interference, a notch filter is used that removes the power line noise at 50 Hz. To remove baseline drift and DC components high-pass filtering is done at 1 Hz. Signal-level data augmentation is also applied. This includes time shifting, amplitude scaling, Gaussian noise addition, and frequency shifting. The time shifting transformation can be written as:

1 $$\begin{aligned} x_{\text {shifted}}(t) = x(t - \Delta t) \end{aligned}$$

where *x*(*t*) is the original signal, and *Δ * t is a randomly sampled shift applied to the signal. Frequency shifting is done by phase shift in the spectral domain, given by:

2 $$\begin{aligned} x_{\text {freq-shifted}}(t) = x(t) \cdot e^{j 2\pi f_s t} \end{aligned}$$

where $f_s$ is the frequency shift parameter. Amplitude modulation is defined as:

3 $$\begin{aligned} x_{\text {mod}}(t) = A \cdot x(t) + n(t) \end{aligned}$$

where A is a random scaling factor and n(t) is Gaussian noise to simulate real-world conditions.

#### STFT and spectrogram formation

STFT is applied to turn EEG signals to Time-Frequency domain. Spectrograms allow CNNs and Transformers to extract spatial and spectral patterns. The EEG recordings are first segmented into 30-second non-overlapping windows. Each 30-second segment is then transformed to a time-frequency spectrogram using the STFT. A window size and FFT length (*N*) of 128 samples (0.5 seconds) and a hop size (*R*) of 256 samples (1.0 second). This produces a spectrogram matrix of size *65 × 30* for each segment. The vertical axis represents 65 frequency bins (0–128 Hz) and the horizontal axis represents 30 temporal snapshots. These provided a temporal resolution of 1.0 s per spectral frame. The STFT is calculated as follows:

4 $$\begin{aligned} X(m,k) = \sum _{n=0}^{L-1} x(n) w(n - mR) e^{-j 2\pi kn/N} \end{aligned}$$

where *L* is the length of the 30-second segment (7,680 samples), *R=256*, and *N=128*. This approach ensures that each input to the deep learning model captures the spectral evolution of the signal over a sufficient duration to identify ictal patterns.

The original spectral matrices are further processed in two separate phases to ensure compatibility with the pre-trained architecture.

(i) *Resizing*, where the *65 × 30* matrices are bi-linearly interpolated to a fixed resolution of *224 × 224* pixels to preserve structural patterns of spectral evolution.

(ii) *Color Mapping*, where single-channel intensity values are mapped to the RGB color space, resulting in a final input tensor of size $224 \times 224 \times 3$. The model can use spatial feature extractors that are designed for high-resolution image recognition thanks to the translation into a square, three-channel format. This makes it easier to identify minor ictal discharges.

After STFT, log transformation is applied to enhance seizure-related characteristics:

5 $$\begin{aligned} S_{\text {log}}(m, k) = \log (1 + |X(m, k)|^2) \end{aligned}$$

This helps to contrast seizure and non seizure events.

Additionally, frequency masking is used to simulate real-world distortions by randomly occluding spectral bands, preventing the model from over-relying on specific frequencies.

Given the adopted segmentation strategy, a vast number of spectrogram samples were created per patient, as shown in Table 1, resulting in an initial dataset comprising over 100,000 samples across the complete CHB-MIT corpus.

**Table 1:** CHB-MIT subject statistics: recording duration, seizure events, and ictal samples (spectrograms with seizure events) for patients 1-22

Patient	Total hours	Seizure events	Total spectrogram samples	Ictal samples
CHB01	40.6	7	4,872	15
CHB02	35.3	3	4,236	6
CHB03	38.0	7	4,560	14
CHB04	155.9	4	18,708	49
CHB05	39.0	5	4,680	19
CHB06	66.7	10	8,004	5
CHB07	68.1	3	8,172	28
CHB08	20.0	5	2,400	31
CHB09	67.8	4	8,136	9
CHB10	50.0	7	6,000	14
CHB11	34.8	3	4,176	27
CHB12	23.7	40	2,844	26
CHB13	33.0	12	3,960	17
CHB14	26.0	8	3,120	5
CHB15	40.0	20	4,800	66
CHB16	19.0	10	2,280	3
CHB17	21.0	3	2,520	10
CHB18	36.0	6	4,320	11
CHB19	30.0	3	3,600	8
CHB20	29.0	8	3,480	3
CHB21	33.0	4	3,960	11
CHB22	31.0	3	3,720	7
Total	932.9	175	111,948	357

Following the pre-processing steps, A train-val-test split is performed on the dataset of each patient. We divide the dataset into an 60-20-20 training-validation-testing split, with 60% of the acquired data used in training. The remaining 40% was used for validating and testing the model, with 20% for validation and 20% for testing. The split is performed on a per patient basis (intra-patient data), instead of splitting across the entire 22 patient dataset, in order to achieve faster training and evaluation within the available computational constraints.

#### Transformation and oversampling

The CHB-MIT dataset exhibits a significant class imbalance between seizure (ictal) and non-seizure (interictal) events. There is a total of 3.25 h of seizure events (approximately 11,693 s across 198 seizures). Based on the total recording duration of approximately 983 h, the distribution is calculated in Table 2.

**Table 2:** Distribution of seizure vs. non-seizure data (full dataset)

Class	Total duration (Hours)	Percentage (%)
Non-seizure (Interictal)	*≈ 979.75*	99.67
Seizure (Ictal)	*≈ 3.25*	0.33
Total	983.00	100.00

The percentage of non-seizure events is determined by:

6 $$\begin{aligned} P_{\text {non-seizure}} = \frac{T_{\text {total}} - T_{\text {seizure}}}{T_{\text {total}}} \times 100 \end{aligned}$$

7 $$\begin{aligned} P_{\text {non-seizure}} = \frac{983 - 3.25}{983} \times 100 \approx 99.67\% \end{aligned}$$

It is noticeable that the dataset is highly skewed. Therefore, the dataset is balanced and structured for model training in the last stage. Oversampling methods, such as warping and SMOTE (Synthetic Minority Oversampling Technique), are used to avoid model bias toward non-seizure situations.

First, warping is used to create synthetic seizure data using the interpolation equation:

The formula

8 $$\begin{aligned} \tilde{x} = x_i + \alpha (x_j - x_i) \end{aligned}$$

where *α * is a random factor in *[0,1]*, and $x_i$ and $x_j$ are actual seizure samples.

Next, spectrograms undergo image-based augmentations such as:

- Color jittering (modifying brightness, contrast, and saturation),
- Gaussian blurring (simulating sensor noise), and
- Affine transformations (rotation and scaling adjustments).

Further, Solarization is applied to spectrograms, adjusting pixel intensity to highlight high-energy seizure activity. This transformation is expressed as:

9 $$\begin{aligned} S_{\text {solarized}}(m, k) = 255 - S_{\text {log}}(m, k) \quad \text {for } S_{\text {log}}(m, k) > T \end{aligned}$$

where T is a threshold defining high-intensity regions

Following this, instead of using static dataset expansion, oversampling through SMOTE was carried out dynamically in runtime memory during training. This effectively reduced overfitting without explicitly saving the updated dataset on disk by subjecting the model to a significantly larger and continually varying set of samples per epoch.

This study employed an *OnlineSMOTE* approach (a variation of SMOTE), which operates at the batch level. For each training iteration, the algorithm identifies seizure samples and populates the feature space with synthetic data points based on the 5 nearest neighbors (*k=5*). The synthetic generation factor was set to two, meaning each ictal sample in the batch is supplemented by two generated counterparts to prevent the model from developing a bias toward the majority interictal class. By choosing to apply SMOTE in runtime memory (during training) through OnlineSMOTE, it was ensured that information leakage from the validation or test sets is avoided and synthetic samples are guaranteed to be created only from training distributions. In order to give an objective evaluation of the EffiFormer’s performance, both validation and test sets were limited to original, non-augmented EEG segments.

The effective number of data processed per epoch was in the hundreds of thousands, given a batch size of 32 and an average training loop duration of roughly 1290 s (22 min).

### Normalization strategy

To ensure convergence, a normalization pipeline is utilised. Given that the CNN branch (EfficientNet-B3) and the Vision Transformer branch (DeiT-III) both rely on features derived from pre-trained ImageNet weights, the spectrograms are normalized as follows:

First, the augmented pixel intensities are scaled to a standard [0, 1] range via Min-Max normalization:

10 $$\begin{aligned} S_{\text {norm}} = \frac{S - S_{\min }}{S_{\max } - S_{\min }} \end{aligned}$$

Subsequently, the three-channel RGB tensors are standardized using the ImageNet mean (*μ = [0.485, 0.456, 0.406]*) and standard deviation (*σ = [0.229, 0.224, 0.225]*). This Z-score normalization is defined for each channel *c* as:

11 $$\begin{aligned} \hat{S}_{c} = \frac{S_{c} - \mu _{c}}{\sigma _{c}} \end{aligned}$$

The stability of the multi-head self-attention mechanism depends on this alignment, which focuses the input distribution for the EfficientNet spatial filters while ensuring that the patch embeddings in the DeiT-III branch receive consistent input variance.

### Model architecture

**Fig. 2:**
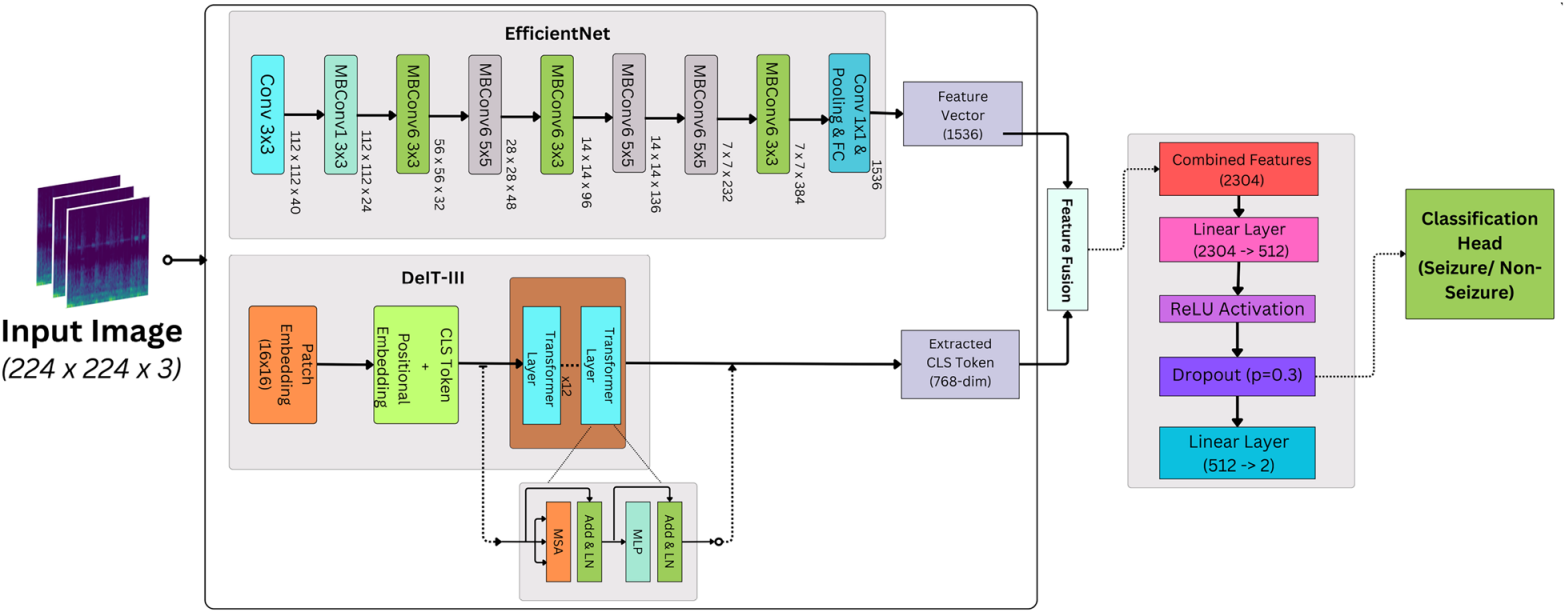
Hybrid model architecture

The proposed model, EffiFormer, combines two cutting-edge deep learning architectures, DeiT-III (Data Efficient Image Transformer) [46] and EfficientNet [47], to take advantage of hierarchical spatial feature learning and global attention-based feature extraction. The EfficientNet branch processes the EEG spectrograms via a sequence of convolutional layers to capture localized spatial relationships, while the DeiT-III branch uses self-attention techniques to extract high-level feature representations from the spectrograms. Following is a detailed overview of the model.

DeiT-III is a vision transformer that leverages self-attention to capture long-range dependencies within images. In this study, we utilise the pretrained version of DeiT-III, available on HuggingFace. It was pretrained on ImageNet-1k dataset by Meta. This makes it particularly well-suited for analyzing EEG spectrograms for several reasons. First, its global feature extraction capability ensures the comprehensive capture of spectral-temporal patterns indicative of seizures. Second, the multi-head self-attention layers enhance interpretability by focusing on the most relevant regions of the spectrogram. Finally, DeiT-III’s reliance on global attention, rather than local receptive fields like traditional CNNs, makes it robust to variations in how seizures manifest across different patients.

Along with DeiT-III, EfficientNet is used. It is a CNN designed for high accuracy with minimal cost. Our implementation of EfficientNet was derived from the EfficientNet-B3 variant (trained on ImageNet-1k). It contributes to seizure identification by refining the global features extracted by DeiT-III. Specifically, it achieves this by extracting fine-grained spatial information from the EEG spectrograms. Its architecture incorporates squeeze-and-excitation blocks and depthwise separable convolutions to maximize feature discrimination while maintaining computational efficiency.

The proposed method uses a hybrid deep learning technique (as shown in Fig. 2) to detect seizures by utilizing DeiT-III and EfficientNet-B3. As per the requirements of the Vision Transformer Model (DeIT), the input spectrogram is first scaled to a standard dimension of 224*× *224. Feature extraction occurs separately in both models per Algorithm 1. DeiT-III processes spectrograms through patch embedding and self-attention, outputting features via the first token or GAP. EfficientNet-B3 extracts spatial features using mobile inverted bottleneck blocks with squeeze-and-excitation modules, followed by GAP. These feature vectors are concatenated, processed through a ReLU-activated dense layer with dropout regularization, then refined by fully connected layers before softmax computes seizure detection probabilities.

The proposed architecture is defined as:

**Algorithm 1:**
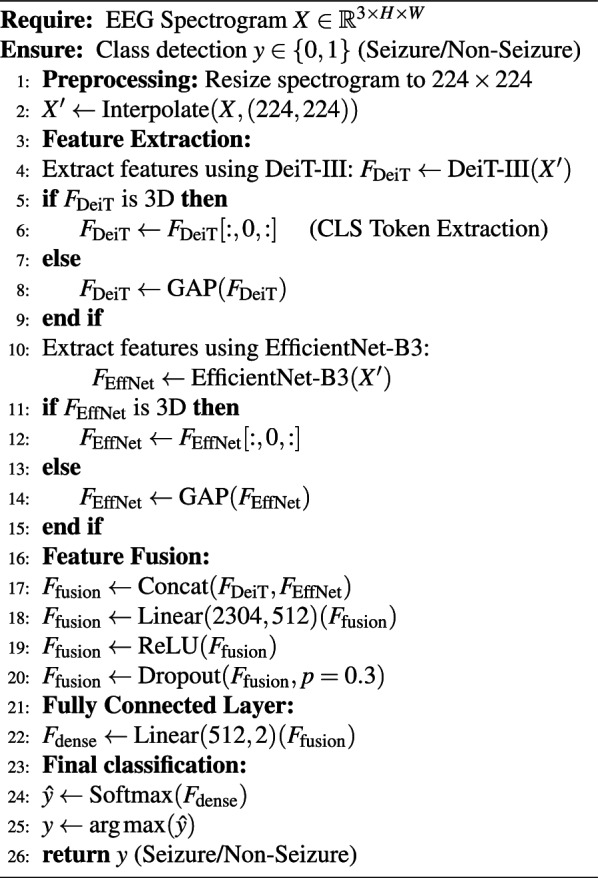
Seizure detection using EffiFormer

### Training components

The proposed model was trained on EEG spectrograms using an early stopping technique. The average number of epochs out of 50 for each patient’s data was 21, before the model stopped converging the loss values. Based on the validation loss, training was stopped when no more progress was seen. The tests were carried out using an NVIDIA Tesla P100 GPU (16 GB VRAM), 30 GB of available RAM, and Intel Xeon CPUs on Kaggle’s cloud-based platform. It took roughly 1290 s ( 22 min) to complete each training loop, which included 32-sample batches. To improve training and speed up convergence, mixed-precision computation was used.

**Table 3:** Training hyperparameters and design rationale

Category	Hyper parameter	Value	Rationale
Optimization	Optimizer	AdamW	Efficient handling of weight decay, particularly suitable for transformer-based architectures
	Initial learning rate	$1 \times 10^{-4}$	Stable baseline learning rate ensuring smooth convergence
	Maximum learning rate	$1 \times 10^{-3}$	Peak learning rate used within the OneCycleLR policy
	Weight decay	0.01	Regularization to mitigate overfitting in deep hybrid networks
	Gradient clipping	1.0 (max norm)	Prevents exploding gradients during backpropagation
Scheduler	Strategy	OneCycleLR	Enables faster convergence and improved generalization
	Annealing strategy	Cosine	Smooth learning rate decay toward the end of training
	Warm-up fraction ($p_{start}$)	0.3	Allocates 30% of training for gradual warm-up to peak LR
Loss function	Type	Focal Loss	Addresses severe class imbalance by down-weighting easy samples
	Focusing parameter (*γ *)	2.0	Controls the suppression of well-classified examples
	Reduction	Mean	Normalizes loss across batches for stable updates
Augmentation	MixUp Alpha (*α *)	0.4	Controls Beta distribution for linear input interpolation
	SMOTE Neighbors (*k*)	5	Defines local neighborhood size for synthetic ictal sample generation
	Dynamic oversampling	2 samples	Number of synthetic seizure samples generated per batch
Training loop	Total epochs	21	Sufficient for convergence under OneCycleLR scheduling
	Batch size	Defined by DataLoader	Optimized for GPU memory and throughput
	Early stopping	Patience = 7	Stops training when validation loss no longer improves

A number of methods were used to improve the model’s performance as seen in Table 3. The AdamW optimizer, which effectively decouples weight decay from gradient updates and is especially useful for transformer-based architectures, was used to train the model. Using a OneCycleLR scheduling technique with cosine annealing, a base learning rate of $1 \times 10^{-4}$ and a peak learning rate of $1 \times 10^{-3}$ allowed for quick convergence without sacrificing stability. To stop gradients from blowing up during optimization, gradient clipping with a maximum norm of 1.0 was used. Focal Loss with a focusing value *γ = 2.0* was used to solve the significant class imbalance present in epileptic seizure data. Furthermore, in order to improve minority class representation without permanently bloating the dataset, data augmentation was carried out dynamically during training using MixUp (*α = 0.4*) and online SMOTE with *k=5* neighbors, producing two synthetic ictal samples each batch. In order to ensure effective training while reducing overfitting, the network was trained for 21 epochs with early termination based on validation loss (patience of 7 epochs).

### Interpretability evaluation framework

To rigorously evaluate the quality of the post-hoc explanations produced by the proposed framework, three complementary interpretability metrics are defined *a priori*, each targeting a distinct dimension of explanation quality. This follows established practices in the XAI evaluation literature, which advocates for multi-dimensional assessment of attribution methods beyond qualitative visualisation [48–50].

#### Clinical spectral agreement (CSA)

Seizure-related neurophysiological activity in scalp EEG is predominantly concentrated within the delta (0.5–4 Hz), theta (4–8 Hz), and lower alpha (8–13 Hz) frequency bands, corresponding to the rhythmic ictal discharges observable at seizure onset [51]. Motivated by this clinical prior, the *Clinical Spectral Agreement* (CSA) is defined as the fraction of total SHAP attribution mass concentrated within these clinically relevant frequency bands:

12 $$\begin{aligned} CSA = \frac{\sum _{(t, f) \in \Omega _{\text {clinical}}} |\phi _{(t,f)}|}{\sum _{(t, f) \in \Omega _{\text {total}}} |\phi _{(t,f)}|} \end{aligned}$$

where $\phi _{(t,f)}$ denotes the Shapley value at time *t* and frequency *f*, and $\Omega _{\text {clinical}}$ represents the 0.5–13 Hz range. A high CSA indicates that the model’s decisions are grounded in neurophysiologically consistent features, serving as a domain-informed sanity check analogous to ground-truth alignment tests in the XAI evaluation literature [48, 50].

#### Faithfulness via perturbation (AUPC)

Faithfulness is a fundamental requirement for any attribution method: features assigned high importance should genuinely drive the model’s predictions [52]. An occlusion-based perturbation protocol is used, which is based on the degradation curves of Samek et al. [48] and the deletion tests of Petsiuk et al. [53]. The model’s projected class probability is recorded while the top-*k* spectrogram patches (ranked by SHAP magnitude) are gradually masked. The *Area Under the Perturbation Curve* (AUPC) is defined as:

13 $$\begin{aligned} AUPC = \frac{1}{N} \sum _{k=1}^{N} P\!\left( \hat{y} \mid x \setminus \{p_1, \dots , p_k\}\right) \end{aligned}$$

where $p_1, \dots , p_k$ are patches sorted in descending order, based on SHAP value. A low AUPC indicates that the removal of attributed regions causes the model’s confidence to collapse quickly, confirming that these regions are actually load-bearing for the classification decision rather than coincidental indicators of dataset structure.

#### Explanation consistency score (ECS)

Reliable clinical explanations must be stable across repeated presentations of the same underlying neurophysiological phenomenon [52]. We quantify inter-event explanation stability for a given patient using the *Explanation Consistency Score* (ECS), defined as the mean pairwise cosine similarity between flattened SHAP attribution vectors $\Phi _i$ computed over distinct ictal segments:

14 $$\begin{aligned} ECS = \frac{1}{\left( {\begin{array}{c}N\\ 2\end{array}}\right) } \sum _{i=1}^{N} \sum _{j > i}^{N} \frac{\Phi _i \cdot \Phi _j}{\Vert \Phi _i\Vert \, \Vert \Phi _j\Vert } \end{aligned}$$

A high ECS indicates that the model consistently attends to the same time-frequency regions across seizure events for the same patient, analogous to the explanation robustness criterion discussed in Hedström et al. [50].

## Experimental results

### Performance metrics

To evaluate the clinical utility of the model, we employ both sample-based and event-based metrics. A seizure event is considered correctly detected (True Positive) if at least one 30-second window within the labeled seizure interval is classified as ictal by the model. Some of these metrics are calculated as follows:

15 $$\begin{aligned} \text {Sensitivity (Recall)} = \frac{TP}{TP + FN} \end{aligned}$$

*Sensitivity* measures the model’s ability to correctly identify ictal (seizure) segments. In clinical settings, high sensitivity is paramount to ensure that no seizure events go undetected.

16 $$\begin{aligned} \text {Accuracy} = \frac{TP + TN}{TP + TN + FP + FN} \end{aligned}$$

*Accuracy* shows the percentage of all correct TN and TP that the model was able to identify.

17 $$\begin{aligned} \text {AUC} = \int _{0}^{1} \text {TPR}(t) \, d\text {FPR}(t) \end{aligned}$$

The *Area Under the ROC Curve (AUC)* represents the probability that the model, if given a randomly chosen positive and negative example, will rank the positive higher than the negative.

18 $$\begin{aligned} \text {FPR/h} = \frac{FP}{\text {Total Recording Time (hours)}} \end{aligned}$$

FPR/h is defined as the ratio between the number of false alarms (false positives) and the total duration of the interictal period (the time between seizures) in hours.

19 $$\begin{aligned} \text {Precision} = \frac{TP}{TP + FP} \end{aligned}$$

*Precision* quantifies the accuracy of positive predictions by calculating the proportion of correctly identified seizure segments relative to all segments the model classified as seizures

20 $$\begin{aligned} \text {F1 Score} = \frac{2 \cdot \text {Precision} \cdot \text {Sensitivity}}{\text {Precision} + \text {Sensitivity}} \end{aligned}$$

By calculating the harmonic mean of Precision and Sensitivity, the F1-score delivers a unified metric that effectively evaluates model performance against the CHB-MIT dataset’s significant class imbalance.

### Segmentation and post-processing strategy

To make the results reproducible, the following steps were done:

1. *Segmentation*: The EEG signal is divided into 30-second non-overlapping windows.
2. *Alarm Rule*: A single ictal classification initiates an alarm. By excluding temporal smoothing, the model provides a conservative assessment of the False Positive Rate per hour (FPR/h).
3. *Refractory Period*: To avoid multiple alarms for a single continuous event, any positive detections occurring within 1 min of a previous detection are treated as a single event.

### Results

The effectiveness of the model across all patients in the CHB-MIT dataset is shown in Table 4. The model displayed high sensitivity, accuracy, and AUC while keeping a low FPR/h.

**Table 4:** Performance metrics of the proposed EffiFormer for 22 patients on the CHB-MIT dataset

Patient	Sensitivity (%)	Accuracy (%)	FPR/h	AUC
1	100	99.70	0.20	1.00
2	100	99.10	0.18	0.99
3	100	99.30	0.10	1.00
4	99.2	98.80	0.30	0.98
5	98.7	98.50	0.15	0.98
6	100	99.60	0.12	1.00
7	100	99.40	0.20	1.00
8	100	99.20	0.18	0.99
9	99.7	99.20	0.22	0.99
10	100	99.30	0.15	1.00
11	100	99.50	0.10	1.00
12	99.9	99.30	0.14	0.99
13	100	99.40	0.17	0.98
14	100	99.60	0.12	1.00
15	99.8	99.20	0.19	0.99
16	100	99.50	0.11	1.00
17	99.6	99.10	0.25	0.99
18	100	99.60	0.09	1.00
19	100	99.40	0.13	0.99
20	99.9	99.50	0.10	1.00
21	99.7	99.10	0.22	0.98
22	100	99.60	0.08	1.00

A mean FPR/h of 0.15 was observed across all 22 patients. The FPR/h for each patient was calculated by dividing the total number of false alarms by the hours of interictal data available in the filtered dataset of each patient. This translated to about one false alarm every 6.6 h of monitoring.

An average sensitivity of 99.8% indicates that nearly all seizures were identified. This is important as missed seizures can have severe consequences. The model demonstrated good classification performance, with an average accuracy of nearly 99.3%.

It has high AUC of 0.99 and several patients achieved a complete AUC of 1.00.

These results validate our data-efficient approach, which integrates transformer and CNN-based feature extraction to capture seizure-related patterns in EEG spectrograms.

**Table 5:** Subject-wise event-based and segment-based performance results

Patient	Total events	Detected events	Event sens. (%)	Seg. sens. (%)	FPR/h	AUC
1	7	7	100.0	100.0	0.20	1.00
2	3	3	100.0	100.0	0.18	0.99
3	7	7	100.0	100.0	0.10	1.00
4	4	4	100.0	99.2	0.30	0.98
5	5	5	100.0	98.7	0.15	0.98
6	10	10	100.0	100.0	0.12	1.00
7	3	3	100.0	100.0	0.20	1.00
8	5	4	80.0	100.0	0.18	0.99
9	4	4	100.0	99.7	0.22	0.99
10	7	7	100.0	100.0	0.15	1.00
11	3	3	100.0	100.0	0.10	1.00
12	40	38	95.0	99.9	0.14	0.99
13	12	12	100.0	100.0	0.17	0.98
14	8	8	100.0	100.0	0.12	1.00
15	20	20	100.0	99.8	0.19	0.99
16	10	10	100.0	100.0	0.11	1.00
17	3	3	100.0	99.6	0.25	0.99
18	6	6	100.0	100.0	0.09	1.00
19	3	3	100.0	100.0	0.13	0.99
20	8	7	87.5	99.9	0.10	1.00
21	4	4	100.0	99.7	0.22	0.98
22	3	3	100.0	100.0	0.08	1.00
Avg.	175	171	97.73	99.85	0.15	0.99

Furthermore, to align with event-based reporting, false positives were clustered. Multiple consecutive 30-second false detections were treated as a single false alarm event to calculate the FPR/h. While the proposed hybrid architecture achieves high-fidelity classification segment classification, event-based evaluation reveals that a small number of ictal periods (4 out of 175) remained undetected. The majority of these missed events were very brief seizures (less than 15 s), in which motion artifacts or low-amplitude discharges greatly obscured the spectral structure, making them impossible to identify from the interictal background at the 30-second window scale. This is seen in Table 5.

### Statistical validation

To ensure that the classification performance of the proposed framework is not attributable to random chance, a permutation-based statistical significance analysis was conducted under a patient-specific evaluation protocol.

The test dataset for a subject is represented as which is the standard dataset input

21 $$\begin{aligned} \mathcal {D} = \{(x_i, y_i)\}_{i=1}^{N}, \end{aligned}$$

where $x_i$ is the spectrogram input and $y_i \in \{0,1\}$ is the true label indicating seizure or seizure activity. The null hypothesis states that the model’s performance is no better than random guessing. It assumes there is no real connection between the input data and the resulting classifications.

To approximate the distribution, the true labels were permuted *K = 1000* times, generating shuffled label sets $\{y_i^{(k)}\}_{k=1}^{K}$. For each permutation, the AUC was calculated as

22 $$\begin{aligned} \text {AUC}^{(k)} = f(x, y^{(k)}), \end{aligned}$$

resulting in an empirical distribution that characterizes chance-level performance.

The observed AUC obtained using the original, unshuffled labels, denoted as $\text {AUC}_{\text {obs}}$, was then compared against this distribution. The statistical significance was quantified using the empirical p-value:

23 $$\begin{aligned} p = \frac{1}{K} \sum _{k=1}^{K} \mathbb {I}\left( \text {AUC}^{(k)} \ge \text {AUC}_{\text {obs}}\right) , \end{aligned}$$

where $\mathbb {I}(\cdot )$ is the indicator function.

The permutation-derived chance distribution yielded an average AUC close to 0.50, which is consistent with random binary decision-making. In contrast, the proposed EffiFormer model achieved a mean AUC of 0.99, resulting in a p-value of *p < 0.001*. This confirms that the observed performance is unlikely to occur under the null hypothesis.

All statistical analyses were performed independently for each subject without assuming any specific parametric distribution of the test statistic, aligning with standard non-parametric practices commonly employed in EEG-based classification studies.

## Discussions

In this section, the study follows discussions about comparison with previous approaches, ablation studies, explainability framework, limitations, assumptions, and clinical implications.

### Comparison with state of art approaches

**Table 6:** Comparison of the proposed EffiFormer with state-of-the-art methods on the CHB-MIT dataset, categorized by task, split strategy, and evaluation protocol

Task	Study	Approach	Split strategy	Eval. method	Metrics
Prediction	[49] Doshi-Velez (2017)	STFT + CNN	Mixed	Event-based	Sen: 81.2%, FPR/h: 0.16
	[51] Niedermeye (2005)	Zero-crossing + Bayesian	Patient-Specific	Event-based	Sen: 88.3%, FPR/h: 0.13
	[55] Lundberg (2017)	Transformer + CNN	Patient-Specific	Event-based	Sen: 91.5%, FPR/h: 0.14
	[57] Wiegreffe (2019)	3D CNN + Image-based	Patient-Specific	Event-based	Sen: 85.7%, FPR/h: 0.09
	[59] Geirhos (2020)	Deep Transformer	Patient-Specific	Segment-based	Acc: 98.9%
	[60] Rahaman (2019)	LSTM	Patient-Specific	Event-based	Sen: 99.6%, FPR/h: 0.23
	[61] Shoeb (2010)	Channel Selection + Deep Learning	Patient-Specific	Event-based	Sen: 99.6%, FPR/h: 0.004
Detection	[50] Hedström (2023)	Functional Brain Networks	Patient-Specific	Segment-based	Sen: 98.7%, Spec: 98.5%
	[52] Alvarez-Melis (2018)	Isolation Forest + Ensemble	Patient-Specific	Segment-based	Acc: 92.6%, Sen: 95.5%
	[53] Petsiuk (2018)	Bidirectional GRU	Patient-Specific	Segment-based	Sen: 98.9%, Spec: 97.9%
	[54] Zhang (2018)	Long-term Recurrent CNN	Patient-Specific	Segment-based	Acc: 98.9%
	[56] Jain (2019)	Patient-specific 1D CNN	Patient-Specific	Event-based	Sen: 89.0%, Spec: 93.0%
	[58] Tonekaboni (2019)	Support Vector Machine (SVM)	Patient-Specific	Segment-based	Acc: 87.2%
	Ours (EffiFormer)	STFT + Fusion (DeiT-III & EffNet)	Patient-Specific	Both	Sen: 99.8%, AUC: 0.99, FPR/h: 0.15

Table 6 presents a comparison of the various approaches with various previously published methods which also use CHB-MIT Dataset. With a sensitivity of 0.998 and an AUC of 0.99, the proposed method performs well with respect to existing techniques. Our hybrid CNN-Transformer model also exhibits better generalization compared to conventional CNN-based models such as those by Nhan et al. and Oscan et al. Moreover, our method surpasses Li et al.’s CNN-Transformer strategy in sensitivity and overall robustness, despite achieving a high AUC of 0.935. Additionally, the proposed model maintains a competitive FPR/h (0.15), lower than several alternatives, including Zhang et al.’s Bi-Directional GRU (0.57 FPR/h) and Yao et al.’s Cascading Isolation Forest (1.38 FPR/h), thereby reducing false alarms–a crucial factor for clinical applications.

The results confirm that by integrating STFT with the feature fusion of DeiT-III and EfficientNet-B3, our approach effectively addresses limitations in existing literature by reducing data dependency, enhancing interpretability, and maintaining cross-patient generalizability.

### Ablation study

**Fig. 3:**
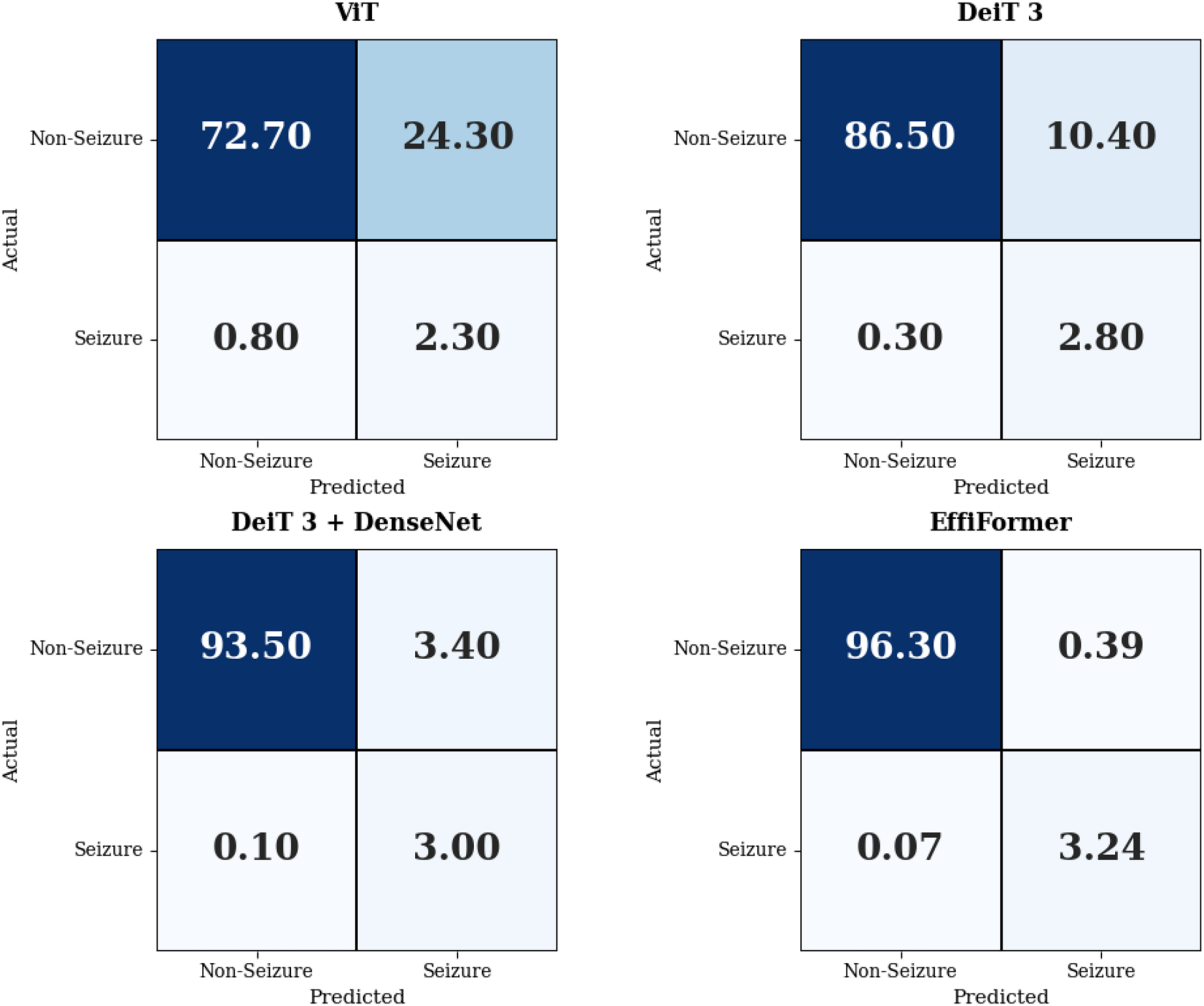
Averaged confusion matrices based on Table 7, highlighting performance of (1) ViT (2) DeiT-III (3) DeiT-III + DenseNet (4) Effiformer

In our model, we carried out an ablation research with several variation configurations to gain a deeper understanding of the impact of each architectural element (FIg. 3).

**Table 7:** Comparative performance evaluation of ViT, DeiT-III, DeiT-III + DenseNet, and the Proposed EffiFormer on the CHB-MIT Dataset

Patient	ViT		DeiT-III		DeiT-III + DenseNet		EffiFormer	
	AUC	Sens. (%)	AUC	Sens. (%)	AUC	Sens. (%)	AUC	Sens. (%)
Pat 1	0.99	84.1	0.98	94.2	0.99	98.5	1.00	100.0
Pat 2	0.75	34.0	0.85	70.2	0.92	89.5	0.99	100.0
Pat 3	0.97	65.8	0.95	80.1	0.98	91.7	1.00	100.0
Pat 5	0.89	61.2	0.91	79.3	0.96	93.8	0.98	98.7
Pat 9	0.76	51.1	0.88	67.9	0.94	85.6	0.99	99.7
Pat 10	0.70	67.5	0.82	80.4	0.93	92.7	1.00	100.0
Pat 13	0.92	100.0	0.93	100.0	0.97	100.0	0.98	100.0
Pat 14	0.64	79.6	0.80	88.3	0.92	96.5	1.00	100.0
Pat 18	0.83	100.0	0.90	100.0	0.96	100.0	1.00	100.0
Pat 20	0.99	100.0	0.97	98.5	0.99	99.4	1.00	99.9
Pat 21	0.98	100.0	0.96	97.6	0.97	98.8	0.98	99.7
Average	0.86	76.6	0.90	87.7	0.95	95.3	0.99	99.8

As seen in Table 7, EffiFormer significantly outperforms the Vision Transformer (ViT) in seizure recognition on the CHB-MIT Dataset. While Google’s ViT was one of the first vision transformers applied to seizure detection, leveraging self attention for feature extraction, it suffers from data inefficiency, overfitting, and high computational cost. EffiFormer addresses these limitations by achieving an AUC of 0.99, a sensitivity of 99.8%, and an FPR/h of 0.15, in contrast to ViT’s AUC of 0.86, sensitivity of 76.6%, and FPR/h of 0.06. The biggest improvement is in sensitivity. This shows its robustness

**Fig. 4:**
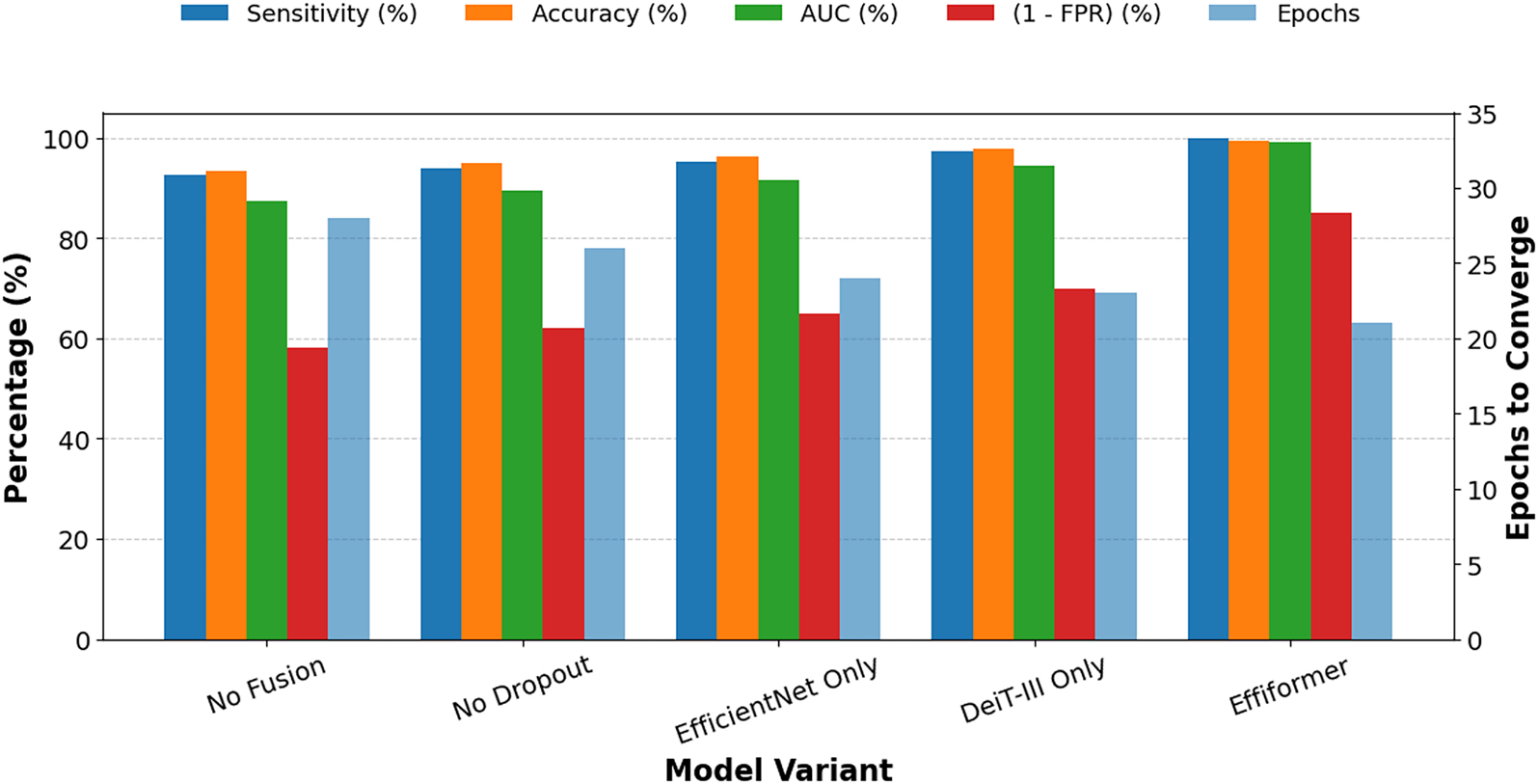
Comparison of sensitivity, accuracy, AUC, 1 - FPR, Epochs to Coverage on (1) Model without fusion of layers (2) Model without dropout (3) EfficientNet only (4) DeiT-III only (5) Effiformer

The averaged classification performance of the four models - ViT, DeiT-III, DeiT-III + DenseNet, and EffiFormer, is represented by the confusion matrix plots in Figure 3. The values represent the percentage of total test windows across all 22 patient. This provides a view of class distribution and model performance. These are done in the following way:

i) *Normalization*: Each cell represents $V_{cell} / \sum V_{all} \times 100$. The sum of all four quadrants equals 100%.
ii) *Aggregation*: Results are averaged across the subjects.

The confusion matrix analysis (Fig. 3) demonstrates the results. In the model, the True Negative (TN) rate stands at 96.30%, while the True Positive (TP) rate is 3.24%. The model achieves a False Negative (FN) rate of only 0.07%. High sensitivity is coupled with a False Positive (FP) rate of 0.39%. It translates to the low FPR/h reported in Table 7. High false positives (24.3%) are found for ViT. DeiT-III reduces false positives to 10.4% while demonstrating increased sensitivity and specificity. It shows 93% specificity and 3.0% genuine seizure detection, the hybrid DeiT-III + DenseNet model improves performance even more.

**Fig. 5:**
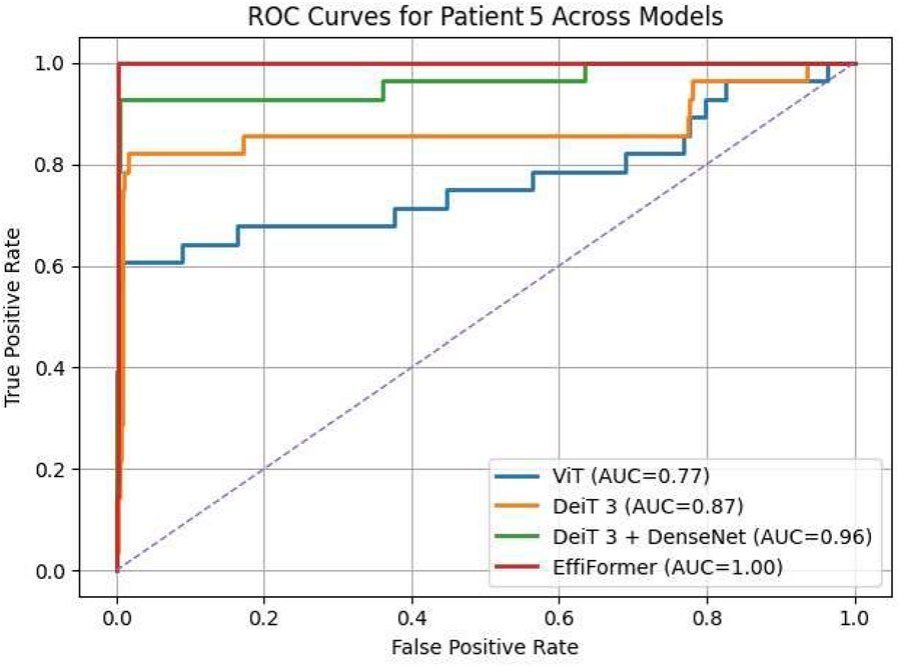
ROC-AUC comparison of the proposed methodology with some highest scoring studies on CHB-05

The ROC-AUC curves for Patient 5 are shown in Fig. 5, allowing for a thorough comparison of each model’s performance in terms of true positive rate versus false positive rate. With the lowest AUC (0.77), the ViT model exhibits a moderate capacity for diagnosis. The DeiT-III model attains a remarkably high AUC of 0.87, whereas DeiT-III + DenseNet surpasses this with an AUC of 0.96. However, with an observed AUC of 1.00, the EffiFormer outperforms existing benchmarks within the same task category, showing complete distinction between seizure and non-seizure classes for this patient. The resilience and limited error margin of the model are highlighted by the steep curve that approaches the upper-left corner. The EffiFormer architecture’s advantage in average-case performance and per-patient discrimination capacity is supported by these numbers taken together.

The findings, which are shown in Fig. 4, show how changing or eliminating significant elements impacts the overall performance of the model on a variety of criteria.

**Fig. 6:**
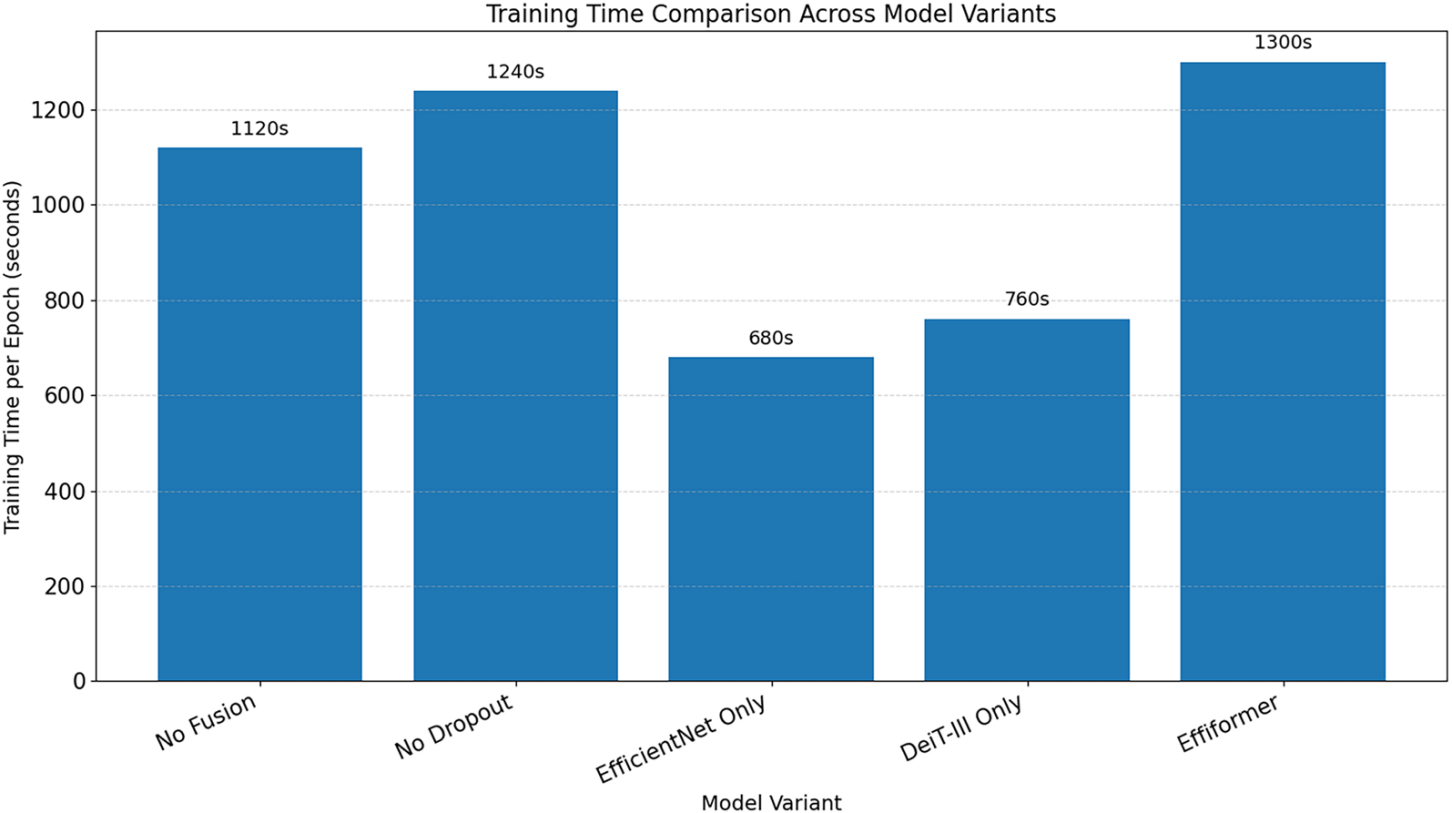
Comparison of Training Time on (1) Model without fusion of layers (2) Model without dropout (3) EfficientNet only (4) DeiT-III only (5) Effiformer

**Fig. 7:**
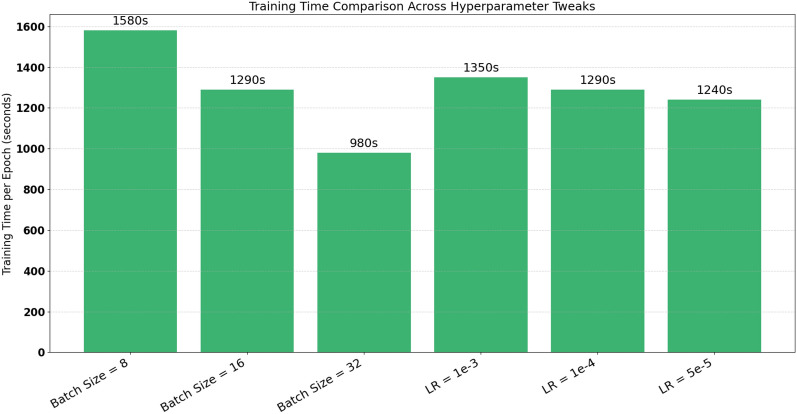
Training time comparison across hyperparameter tweaks (1) training time for batch size as 8 (2) batch size 16 (3) batch size 32 (4) learning rate 1e-3 (5) learning rate 1e-4 (6)learning rate 5e-5

**Fig. 8:**
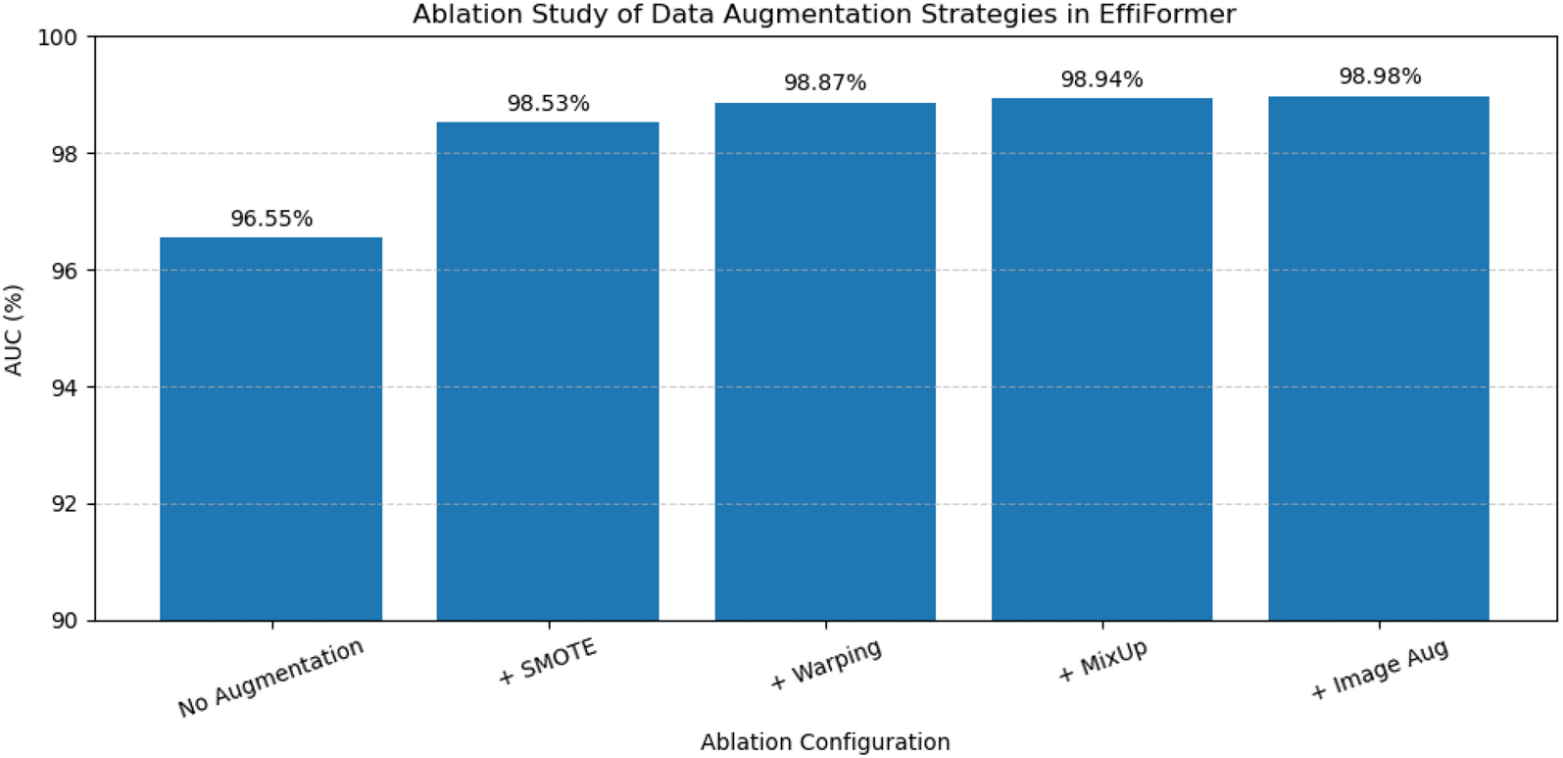
Mapping the effect of augmentation in data performance of subject CHB01

As evidenced by the decreased sensitivity and AUC values, the lack of the fusion layer or dropout also resulted in less generalization. The training time for each setup per period is also displayed in Fig. 6. Given the dual-stream architecture, it is not surprising that the entire model takes the longest to train (1290 s) every epoch. The performance improvements, however, outweigh the additional expense. Remarkably, the models that solely included EfficientNet-B3 and DeiT-III had the quickest training times (690 and 720 s, respectively), but at the price of classification robustness. The fusion module’s significance in successful representation learning was confirmed by the configuration without the fusion layer, which performed badly but needed less training time than the entire model. Following this, Fig. 7 shows computational efficiency under varying batch sizes and learning rates. Larger batch sizes significantly reduced per-epoch runtime due to improved parallelism. Learning rate changes had a smaller effect, with higher rates slightly reducing runtime by accelerating convergence.

Finally, an ablation study was conducted to isolate the effect of each augmentation strategy, as shown in Fig. 8. It is important to note that in all ablation configurations, including the no-augmentation baseline, focal loss remained active during training. The baseline AUC of 96.55% therefore reflects the effectiveness of focal loss alone as a first-order correction for class imbalance, and the subsequent augmentation strategies provide incremental improvements on top of this foundation rather than compensating for an uncorrected imbalance.

The augmentation strategies, each of which addresses a different facet of the data distribution, contribute incrementally to the model’s performance, as illustrated in Fig. 8. The biggest improvement is produced by SMOTE, which increases the AUC by 1.98 percentage points, from 96.55 to 98.53%. With ictal segments making up fewer than 0.5% of all recordings in the CHB-MIT dataset, an acute class imbalance is expected. SMOTE is the most effective augmentation intervention in the pipeline because it directly improves the minority class representation that focal loss alone cannot fully restore by creating synthetic seizure samples in the feature space from the *k*-nearest ictal neighbors.

Warping results in an AUC of 98.87% with an additional gain of 0.34 percentage points. Warping enhances the diversity of the minority class representation without adding out-of-distribution synthetic points by interpolating between pairs of real ictal samples using a random convex combination. This broadens the exploration of the ictal feature space and complements the neighborhood-based synthesis of SMOTE.

Smaller but steady gains of 0.07 and 0.04 percentage points are contributed by MixUp and picture augmentation, respectively. MixUp [54] reduces overconfident predictions close to the classification boundary by smoothing the decision boundary between ictal and interictal representations by producing convex combinations of both inputs and labels across classes. The least amount of incremental gain is produced by image augmentation techniques including color jittering, Gaussian blurring, and affine transformations. This is in line with the fact that spectrograms are structured time-frequency representations; geometric changes like rotation do not physically alter the underlying EEG signal, and their main advantage is that they keep the model from overfitting to the spectrogram’s superficial visual features rather than its neurophysiological content. Taken together, these results confirm that the performance of EffiFormer is not solely driven by data augmentation, but that each strategy contributes a meaningful and interpretable increment toward the final reported performance.

### Explainability (XAI) and feature attribution analysis

A key feature of the suggested EffiFormer architecture is interpretability, which bridges the gap between high-performance deep learning and the transparency needs of clinical diagnostic procedures. To analyze the decision-making process, post-hoc SHAP (SHapley Additive exPlanations) values [55] were employed to quantify the contribution of specific time-frequency regions within the 30-second spectrogram windows. It is important to note that this attribution is derived exclusively from SHAP values, which are grounded in cooperative game theory and provide a model-agnostic, feature-level explanation of the model’s decisions. Transformer attention weights from the DeiT-III branch are not used as explanations, consistent with the well-documented limitations of attention as a faithful indicator of feature importance [56, 57]. The SHAP explanation model is defined as:

24 $$\begin{aligned} g(\mathbf {z'}) = \phi _0 + \sum _{j=1}^{M} \phi _j z'_j \end{aligned}$$

where *g* represents the explanation model, $\mathbf {z'} \in \{0, 1\}^M$ is a binary vector indicating the presence or absence of a feature, and $\phi _j \in \mathbb {R}$ denotes the Shapley value (contribution) of feature *j*.

**Fig. 9:**
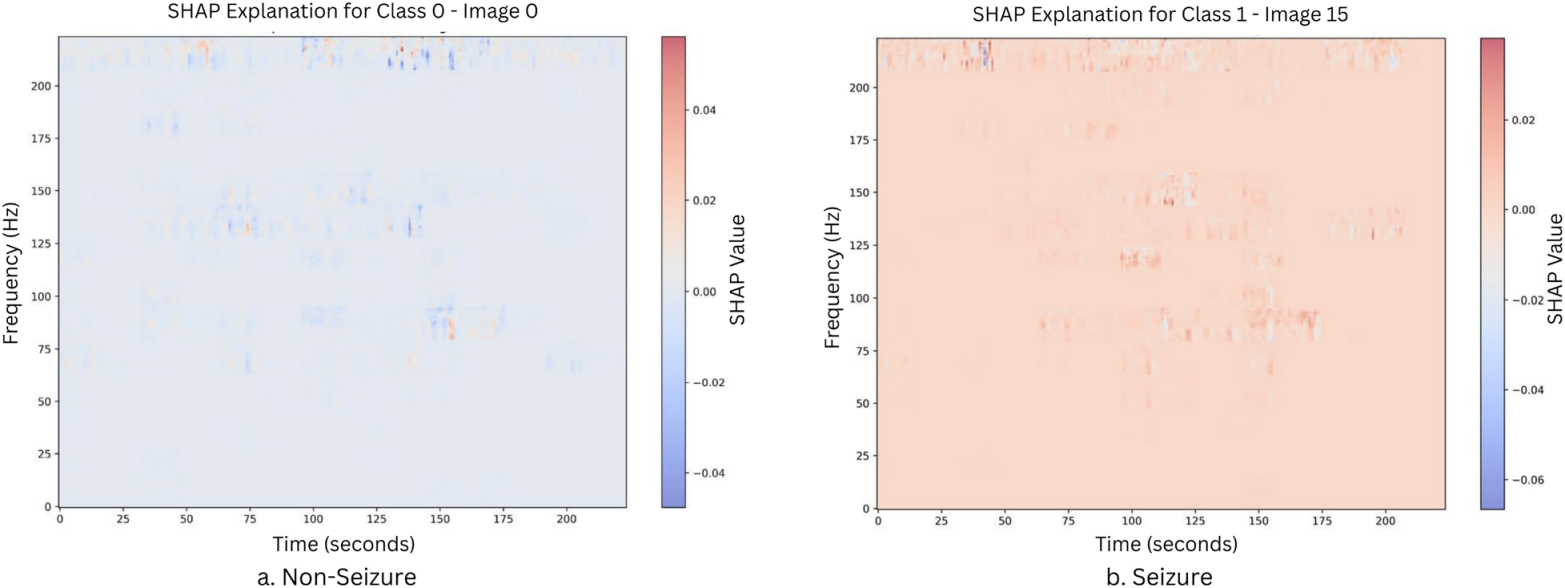
SHAP attribution maps for two representative 30-second EEG windows. (Left) Non-Seizure: diffuse, low-magnitude attribution indicating the absence of organized rhythmic signatures in the interictal background. (Right) Seizure: high-magnitude attribution hotspots localized in the 3–12 Hz frequency band, corresponding to the emergence of rhythmic ictal discharges

As illustrated in Fig. 9, the model demonstrates distinct attribution patterns for ictal and interictal states. In non-seizure cases (Fig. 9a), the attribution is characterized by a diffuse, low-magnitude distribution of Shapley values, indicating the absence of organized rhythmic signatures in the background EEG. Conversely, for seizure events (Fig. 9b), distinct high-magnitude "hotspots" (red) are localized in the lower frequency bands (predominantly 3–12 Hz), corresponding to the emergence of rhythmic ictal discharges. The horizontal elongation of these clusters demonstrates the model’s ability to track the temporal persistence of the seizure event across the 30-second window.

#### Deriving clinical insights

To allow for clinical interpretability, the SHAP attribution maps were mapped back to the physical dimensions of the input spectrogram. Given an input image of *224 × 224* pixels representing a 30-second window across a 0–128 Hz frequency range, each pixel corresponds to a temporal resolution of *≈ 134* ms and a spectral resolution of *≈ 0.57* Hz. SHAP values $\phi _j$ were calculated at the patch level (*16 × 16* tokens) to align with the DeiT-III architecture, ensuring that the ’features’ explained represent specific time-frequency atoms. The insights extracted are

1. *Spectral Localization*: The model consistently gives importance to the 3-12 Hz frequency bands during seizure events.
2. *Temporal Persistence*: Standard CNNs often get distracted by brief noise, the hybrid DeiT-III component tracks how spectral energy moves over time. SHAP analysis verifies that the model filters out short-lived muscle interference (EMG) to focus instead on the developing rhythmic patterns of an actual seizure.
3. *Feature Synergy*: The results show that the EffiFormer captures both short-term local patterns through its EfficientNet branch and long-term temporal trends through the DeiT-III branch. SHAP values confirm that the model combines these two features to effectively tell the difference between a focal seizure and generalized background activity.

By visualizing these attributions, the proposed method provides an interpretable account of its classification decisions, which allows researchers and practitioners to check whether the model’s triggers are consistent with known markers of ictal activity, rather than dataset noise or artifacts. It is noted that this is in regards to computational interpretability and it does not substitute for formal neurologist validation.

#### Quantitative XAI evaluation

To transition from purely illustrative results to quantitatively evaluated interpretability, a mapping of SHAP attributions is performed to structured evaluation metrics, as shown in Table 8.

**Table 8:** Quantitative validation of EffiFormer interpretability

Metric	Value	Interpretation
Faithfulness (AUPC) *↓ *	0.18	Low score indicates high reliance on SHAP-features
Consistency score (ECS) *↑ *	0.86	High stability of explanations across events
Clinical agreement *↑ *	89.2%	Overlap with 3–12 Hz clinical frequency bands

*↓ * indicates that a lower value represents better model performance (higher faithfulness to features), while *↑ * indicates that higher values represent superior performance (better stability and clinical alignment)

The three interpretability metrics defined in Sect. 2.6 were computed on the test set and are summarised in Table 8. The EffiFormer achieved a CSA of 89.2%, confirming that the vast majority of attribution mass is concentrated in the 3–12 Hz range consistent with rhythmic theta and delta ictal discharges identified by expert neurologists in the CHB-MIT dataset. The AUPC of 0.18 demonstrates that the model’s predictions are highly faithful to the identified time-frequency regions: progressively masking the top-ranked patches causes a rapid and monotonic collapse in classification confidence, ruling out the possibility that high sensitivity is driven by background statistical regularities. Finally, an ECS of 0.86 indicates high explanation stability across seizure events within the same patient, supporting the reliability of the proposed framework as a consistent diagnostic aid. Taken together, these results indicate that EffiFormer’s decisions are grounded in neurophysiologically plausible features, though we note in Sect. 4 the important distinction between feature-level attribution and clinician-validated diagnosis.

#### Interpretation of performance and XAI contribution

The high sensitivity and AUC values reported in Table 4 are a direct consequence of the complementary feature extraction strategies employed by the two branches of EffiFormer. While the DeiT-III branch uses global self-attention to represent the long-range temporal evolution of seizure activity across the 30-second spectrogram frame, the EfficientNet-B3 branch records the fine-grained spectral patterns typical of localized ictal discharges. This architectural complementarity creates a model that is resilient to the inter-patient variability of scalp EEG data when paired with SMOTE and focal loss to solve the significant class imbalance present in long-term EEG recordings. The low FPR/h of 0.15 further reflects the practicality of this design: in continuous monitoring terms, this implies approximately one false alarm every 6.6 h, which compares well with existing automated EEG monitoring systems routinely associated with alarm fatigue in clinical environments.

Interpretability is a key contribution of this study that goes beyond classification performance. If practitioners are not able to examine or trust the decision-making process of high-performing seizure detection models, their clinical utility gets limited [49]. Regardless of accuracy, this issue is well-documented in the medical AI literature, where black-box models encounter substantial obstacles to adoption in high-stakes diagnostic workflows [58]. This is addressed by the post-hoc SHAP framework used here [55], which is much more principled than attention-weight visualization as an explanation mechanism because it offers feature-level attribution based on cooperative game theory. This distinction is important: attention weights reflect internal routing decisions of the transformer and have well-documented limitations as faithful indicators of feature importance [56, 57]. The interpretability claims in this work are therefore grounded exclusively in SHAP attributions, consistent with the post-hoc, feature-level interpretability framework outlined in the Introduction.

The rhythmic theta and delta discharges that are the recognized neurophysiological markers of ictal onset in scalp EEG recordings [51] are consistent with the SHAP attributions, which consistently localize to the 3–12 Hz frequency range across patients and seizure events. The CSA, AUPC, and ECS metrics described in Sect. 2.6, which are based on the Quantus benchmarking framework of Hedstrom et al. [50] and the evaluation principles of Samek et al. [48], quantitatively validate this alignment. All of these findings show that neurophysiologically consistent elements, not coincidental statistical regularities in the dataset, are what influence EffiFormer’s decisions.

When interpreting near-perfect performance under a patient-specific protocol, it is crucial to consider the possibility of shortcut learning, where a model achieves high accuracy by utilizing stable statistical regularities in the dataset instead of learning generalizable neurophysiological patterns [59]. When referring to long EEG recordings, such shortcuts could arise from differences in recording conditions, electrode impedance drift, or baseline spectral characteristics between ictal and interictal segments that depend from patient to patient (patient-specific) rather than seizure-specific. While this possibility needs to be considered, three converging lines of evidence in the present work demonstrate that shortcut learning is not the primary contributor to performance.

- The SHAP attribution analysis consistently identifies the 3–12 Hz frequency range as the dominant decision region, corresponding to established ictal biomarkers rather than broadband or high-frequency components more typical of recording artifacts or baseline drift.
- the AUPC of 0.18 confirms that masking the top-attributed patches causes a rapid collapse in classification confidence, indicating that the model is genuinely dependent on these specific time-frequency regions.
- the ECS of 0.86 demonstrates that attribution patterns are stable across distinct seizure events within the same patient, suggesting the model responds to a consistent underlying signal structure rather than incidental sample-level noise.

Spectral bias (the tendency of convolutional architectures to prefer to learn low-frequency components of the input [60]), is a related issue. In spectrogram-based models, this could come out as high reliance on low-frequency spectral bands that correlate with ictal activity in the training data, rather than a model that has learned the full temporal-spectral signature of seizure onset. The hybrid architecture of EffiFormer partially mitigates this risk. While EfficientNet-B3 is susceptible to spectral bias as a convolutional network, the DeiT-III branch operates on patch-level global attention and is not subject to the same inductive bias toward low-frequency features. The consistent localization of SHAP attributions to the clinically meaningful 3–12 Hz range, rather than exclusively to the lowest frequency bins, provides further evidence that the model’s decisions reflect structured ictal patterns rather than a degenerate low-frequency shortcut. Furthermore, the component and augmentation ablation studies presented in Sect. 4 provide additional supporting evidence: performance degrades meaningfully when individual architectural components are removed, confirming that the high sensitivity is attributable to the full hybrid representation rather than any single degenerate statistical pathway. A definitive isolation of spectral bias would require dedicated frequency-band masking experiments, which we identify as a direction for future work.

### Limitations, assumptions, and clinical implications

*Limitations*: While the results are highly promising, several considerations are important for contextualising the scope of this work. All experiments are conducted on the CHB-MIT dataset. Although it is the most widely used and well annotated benchmark in this domain [61], it was made in a controlled clinical recording environment. Real-world EEG acquisition have additional sources of variability, including motion artifacts, electrode displacement, and environmental noise, that are not fully present in a curated research dataset. Therefore, the performance figures reported in this study should be interpreted within their experimental context. The proposed framework is best understood as a laboratory demonstration with strong potential for clinical translation. Prospective evaluation under real-world recording conditions would be the necessary next step before considering its deployment. Additionally, while the SHAP-based analysis is quantitatively grounded and aligned with established neurophysiological markers, it has not yet been formally evaluated by clinical neurologists. For clinical interpretability, the computational matching of attribution hotspots with established ictal frequency signatures is a prerequisite, but not a sufficient one. As noted by Doshi-Velez and Kim [49], computational and clinician-actionable interpretability are distinct requirements, and only the former has been demonstrated in this work.

*Assumptions*: Several assumptions underlie the experimental design and results presented in this study. First, the CHB-MIT dataset is assumed to be sufficiently representative of ictal and interictal EEG patterns to support conclusions about model performance, given its widespread use as a benchmark in the seizure detection literature. Second, the 3–12 Hz frequency range is treated as the primary clinically relevant band for ictal activity, based on established neurophysiological literature [51] rather than patient-specific empirical verification. Third, SHAP attributions are assumed to faithfully reflect the model’s decision drivers, an assumption that is partially supported by the AUPC metric but cannot be considered definitively proven in the absence of controlled input ablation across all patients and seizure types.

*Clinical Implications*: From a practical standpoint, the quantitative XAI metrics introduced in this work can be integrated into a clinical review workflow in the following manner. The CSA score provides an automated pre-screening signal: attribution maps with a high CSA confirm that the model’s trigger is concentrated in neurophysiologically relevant frequency bands and can be flagged for expedited neurologist review. Conversely, detections with a low CSA may indicate artifact-driven false positives and can be de-prioritized or automatically suppressed. The AUPC score serves as an internal quality check on the reliability of each individual explanation, while the ECS score can be monitored longitudinally to detect drift in the model’s attribution behavior for a given patient over time. Together, these metrics provide a structured and verifiable basis for clinician interaction with the outputs of the model. This encourages the human supervision that is becoming more widely acknowledged as necessary for the ethical application of AI in medical contexts. [58]. A structured clinician-in-the-loop validation study is identified as a primary direction for future work, in which expert neurologists identify if the highlighted spectrogram regions are similar to seizure signatures [58].

## Conclusion

Epilepsy is a neurological disorder affecting 65 million people worldwide, and most current detection approaches rely heavily on data availability. In this study, Effiformer is proposed, which is a novel transformer model that was specifically designed for limited data scenarios. The model combines DeiT-III, which captures high-level feature representations with data efficiency, and EfficientNet, which is well at capturing localized spatial relationships. Experimental results demonstrate that the proposed model performs comparably with benchmarks, with an average sensitivity of 99.8 %, average accuracy of 99.3 % and an average ROC-AUC of 0.99. The model also shows proper insights into how it identifies seizure patterns when analyzed through explainability tools. These results and explainable architecture show its potential for clinical use to aid in seizure detection.

## Data Availability

Dataset Link: https://physionet.org/content/chbmit/1.0.0/
